# Comparison of Immunohistochemical Markers in Oral Submucous Fibrosis and Oral Submucous Fibrosis Transformed to Oral Squamous Cell Carcinoma—A Systematic Review and Meta-Analysis

**DOI:** 10.3390/ijms241411771

**Published:** 2023-07-21

**Authors:** Diksha Mohapatra, Swagatika Panda, Neeta Mohanty, Saurav Panda, Natalia Lewkowicz, Barbara Lapinska

**Affiliations:** 1 Department of Oral Pathology and Microbiology, Institute of Dental Sciences, Siksha ‘O’ Anusandhan University, Bhubaneswar 751003, Odisha, India; 2 Department of Periodontics and Oral Implantology, Institute of Dental Sciences, Siksha ‘O’ Anusandhan University, Bhubaneswar 751003, Odisha, India; 3 Department of Periodontology and Oral Diseases, Medical University of Lodz, 251 Pomorska St., 92-213 Lodz, Poland; 4 Department of General Dentistry, Medical University of Lodz, 251 Pomorska St., 92-213 Lodz, Poland

**Keywords:** oral submucous fibrosis, oral cancer, oral squamous cell carcinoma, systematic review, meta-analysis, biomarkers

## Abstract

The objective of the study was to compare the expression of immunohistochemical (IHC) markers of oral submucous fibrosis (OSMF) (non-transformed group) to those of oral squamous cell carcinoma (OSCC) transformed from OSMF (transformed group). The search for comparative cross-sectional studies was carried out in PubMed and Scopus abiding to the PICO criteria, where expression of IHC markers in OSMF were compared with that of OSCC transformed from OSMF. The cellular distribution, number of positive cases, staining intensity, and mean immunoreactive score (IRS) of each IHC marker were evaluated in both groups. A total of 14 studies were included in the systematic review, in which immunoexpression of 15 epithelial and 4 connective tissue biomarkers were evaluated. Expression of β1-integrin, OCT-3, CD1a, CD207, survivin, Dickkopf-1, COX-2, hTERT, CTGF, MDM2, Ki-67, and α-SMA were increased during transformation of OSMF to OSCC. Conversely, expression of PTEN and lysyl oxidase decreased during transformation of OSMF to OSCC. Expression of a group of epithelial markers, such as COX2, hTERT, CTGF, survivin, MDM2, and p53, was 38 times lower in the non-transformed group cases compared to transformed group cases (95% CI: 58% to 10%; *p* = 0.01; and I^2^ = 90%). Meta-analysis of all markers involved in cell metabolism/apoptosis, which included β1-integrin along with the above markers also suggested 42 times lower expression in the non-transformed group as compared to the transformed group (95% CI: 58% to 10%; *p* = 0.01; and I^2^ = 90%). Sub-group analyses on cytoplasmic and nuclear epithelial markers were inconclusive. Meta-analysis of connective tissue markers was also inconclusive. No publication bias was found. Instead of delving into numerous markers without a strong basis for their use, it is advisable to further study the markers identified in this study to explore their clinical utility.

## 1. Introduction

Oral submucous fibrosis (OSMF) is a chronic, insidious oral potentially malignant disorder (OPMD) that causes fibrosis of tissues, collagen deposition, and scar tissue formation [1,2]. Universally, OSMF is known as a disease of the Asian region, especially predominant in the Indian sub-continent due to high tobacco and areca nut consumption [3]. Progressive OSMF affects oral health, resulting in eating and speaking difficulties. Moreover, individuals with OSMF are at risk of developing oral squamous cell carcinoma (OSCC), with malignant transformation rates ranging from 1.5 to 15% [4]. Mechanisms of malignant transformation of OSMF differ from other OPMDs [5]. This difference might be related to specific carcinogenic properties of areca nut. Specifically, areca alkaloids cause cytotoxic and genotoxic effects on the oral epithelium [6]. Another possibility is that collagen accumulation in the submucosa might lead to tissue hypoxia, which is a cancer-inducing factor [7]. Chronic irritation due to areca nut usage upregulates pro-inflammatory cytokines and reduces anti-fibrotic IFN-gamma [8], which causes increased fibrosis and juxtaepithelial inflammatory reaction, eventually resulting in epithelial atrophy. Thus, multiple molecular events are happening in both epithelium and connective tissue, leading to the malignant transformation of OSMF, which is why there are numerous studies [9,10,11] on site-specific biomarkers in OSMF. Expression of P53 [12,13,14], Ki-67 [15], Bcl2 [16], and Bax [17] in the basal and parabasal layers of epithelium are reportedly high in OSMF and reported as early events of the malignant transformation of OSMF. Invasion and epithelial mesenchymal transition are stimulated by several connective tissue proteins in OSMF that lead to malignant transformation. Vimentin, insulin-like growth factor-1, and fibronectin are a few connective tissue proteins known to be activated in human fibrotic buccal mucosal fibroblasts isolated from OSMF tissues [18,19]. Generally, downregulation of cell junction-specific epithelial markers and upregulation of epithelial mesenchymal transition-specific connective tissue markers are known to initiate malignant transformation of OSMF [18]. Prospective longitudinal study designs can offer a thorough assessment of the predictive potential of immunohistochemical markers by examining these markers in OSMF tissue and re-evaluating them within the same patient after malignant transformation into OSCC occurs. However, such research design is not practically feasible. Instead, cross-sectional studies comparing the immunohistochemical markers in OSMF without malignant transformation (non-transformed group) and OSCC in pre-existing OSMF (transformed group) are feasible. Such research designs have the potential to identify and narrow down the possible immunohistochemical markers responsible for the malignant transformation of OSMF. There are many such studies offering the inconclusive role of the markers on the malignant transformation potential of OSMF [20,21,22,23,24,25,26,27]. Therefore, this systematic review and meta-analysis aimed to evaluate the immunohistochemical biomarkers involved in the transformation of OSMF to OSCC. The objective of this study is to identify and compare the expression differences of immunohistochemical markers between the non-transformed group (OSMF) and the transformed group (OSMF+OSCC). The findings of this review may provide insights into predictive markers for the malignant transformation of OSMF, address knowledge gaps, and offer recommendations for future studies.

## 2. Method

This systematic review and meta-analysis was conducted abiding by the preferred reporting for systematic review and meta-analysis (PRISMA) checklist as described by Moher et al. in 2010 [28]. This systematic review was registered in the PROSPERO database with a registration number CRD42021286558.

### 2.1. Study Design

This systematic review and meta-analysis of human studies was conducted on comparative cross-sectional studies, which analyzed and compared the immunohistochemical expression of markers in non-transformed and transformed OSMF, both qualitatively and quantitatively. Intensity of expression was the qualitative feature, whereas number of immunopositive cases and immunohistochemistry score were the quantitative features.

### 2.2. Search Strategy

PECOS framework was used to construct the search strategy. Patients histopathologically diagnosed with OSMF (non-transformed group) were the population (P) and immunohistochemistry of the tissue was the exposure (E). Patients histopathologically diagnosed with oral squamous cell carcinoma with pre-existing OSMF (transformed group) were compared (C) with the population. The number of immunohistochemically positive cases and the immunohistochemical score were the quantitative outcomes (O). Intensity of immunohistochemical expression in both groups was the qualitative outcome (O). The electronic search was carried out in two databases, PubMed and Scopus, limited to September 2022, to identify the reports to answer the research question.

Search was carried out using both MeSH and keywords using the following phrases: (“oral submucous fibrosis”[Title/Abstract] OR “premalignant condition”[Title/Abstract] OR “precancerous condition”[Title/Abstract] OR “potentially malignant disorder” [Title/Abstract]) AND (“oral squamous cell carcinoma” [Title/Abstract] OR “oral cancer” [Title/Abstract] OR “malignant transformation” [Title/Abstract] OR “oral malignancy” [Title/Abstract]) AND (“immunohistochemistry” [Title/Abstract] OR “immunoexpression”[Title/Abstract] OR “biomarkers” [Title/Abstract] OR “markers” [Title/Abstract]). The reference lists of the selected articles were also searched.

### 2.3. Study Selection

Three reviewers (S.P., D.M., and Sa.P.) independently screened the selected articles, first by title and abstract, followed by full-text considering the inclusion and exclusion criteria. Any disagreement was solved by a fourth reviewer (N.M.). Both retrospective and prospective cross-sectional comparative studies with a well-defined study population and immunohistochemical staining protocol were included. Cross-sectional studies compared immunoexpression in two groups: the non-transformed and transformed groups were included. Studies on biopsy specimens of OPMDs other than OSMF, and biopsy specimens of cancers in sites other than the oral cavity, such as oropharyngeal cancers, nasopharyngeal cancer, esophageal cancers, and metastatic or recurrent carcinomas, were excluded.

### 2.4. Data Extraction

Three investigators (S.P., D.M., and Sa.P.) independently extracted information from the included articles, such as author name, year of publication, population, sample size, age, male–female ratio, location, markers, cellular location of markers, number of positive cases, staining intensity, and mean immunoreactive score in both groups. Quickscore is defined as sum of intensity score and proportion score [26]. Immunoreactive score is derived by multiplying staining intensity with percentage of positive cells [20], labeling index is calculated as (No. of positive cells/No. of cells) × 100 [29], and H-score is calculated as 1 × (% of 1 + cells) + 2 × (% of 2 + cells) + 3 × (% of 3 + cells) [30].

Since there was no uniformity in reporting stages of the non-transformed group, we combined moderately advanced and advanced staging under the advance stage. Quality assessment of all the included studies was conducted as per the Newcastle–Ottawa scale checklist for cross-sectional studies [31]. Disagreements were resolved by discussion with multiple authors (Sa.P., N.M.). The STROBE criteria evaluated all the articles included in the systematic review and meta-analysis [32]. The meta-analysis was conducted in Review Manager Software (REVMAN version 5.4.1). Forest plots were constructed for each reported outcome, risk ratio of number of immunohistochemically positive cases in the non-transformed and transformed groups being the effect measure. Egger’s Test was conducted, in which *p*-value, lower limit, and upper limit of confidence intervals (CI) were assessed to evaluate publication bias in the included studies.

## 3. Results and Discussion

### 3.1. Study Selection

**Figure 1:**
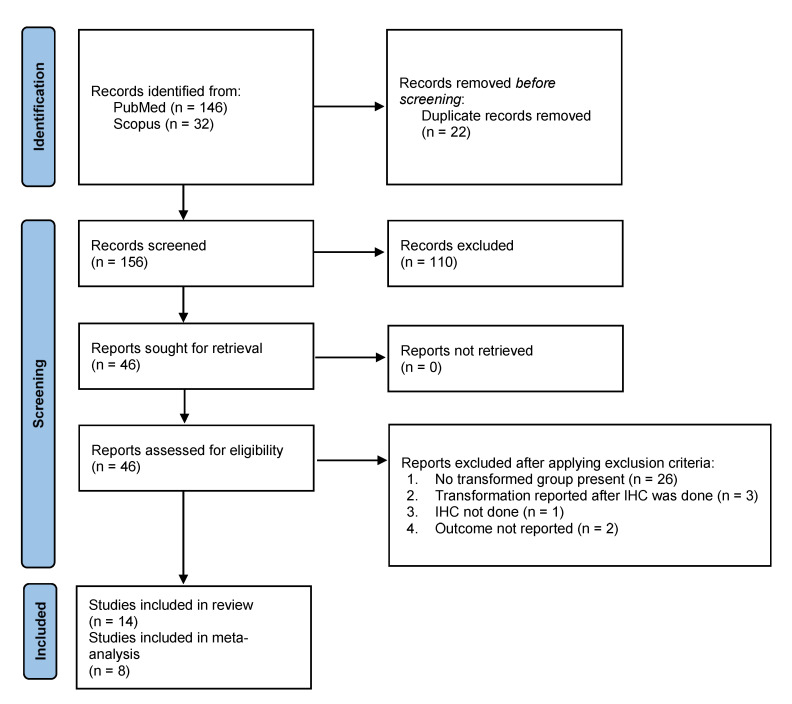
Study selection—PRISMA flowchart.

A total of 178 records were retrieved from PubMed and Scopus database, out of which 156 records were retrieved after duplicate removal. After screening titles and abstracts, 110 records were excluded. Full texts of 46 articles were assessed for complete review. Further comprehensive evaluation against the inclusion criteria excluded 32 articles. The total of 14 articles were included in the systematic review [20,21,22,23,24,25,26,27,29,30,33,34,35,36], as shown in Figure 1.

### 3.2. Study Characteristics

A total of 14 studies are included in the systematic review, in which immunoexpression of 19 biomarkers were evaluated [20,21,22,23,24,25,26,27,29,30,33,34,35,36]. The following biomarkers were studied: survivin [20], hTERT [21], β1 integrin [22], caspase-3 [23], α-SMA [25,29], COX-2 [24], p53 [24,27], MDM2 [24], PTEN [25], CTGF [26], Ki-67 [29,36], CD105 [29], OCT-3 [33], Dickkopf-1 [30], Lysyl oxidase [34], CD1a [35], CD207 [35], CD303 [35], and p16 [36]. Of the total 14 articles, 9 studies were conducted in Indian [21,22,23,24,25,26,29,34,36], 4 in other Asian [20,27,30,33], and 1 in Brazilian population [35]. The non-transformed group and transformed group were comprised of 795 and 637 patients, respectively. Six out of sixteen articles specified the gender of the patients in the non-transformed group, which demonstrated male to female proportion as 227:56 [21,24,25,26,30,35]. In the transformed group, the male–female proportion of the patients was stated in seven articles [20,21,24,25,26,30,35], which included 188 males to 31 females. Eight articles further subdivided the non-transformed group (OSMF) into early and advance stage [20,21,22,23,24,25,26,29]. The site of biopsy in the non-transformed group was reported in only four articles [24,30,34,35], buccal mucosa being the most reported location. Data regarding site of manifestation of the transformed group were available in seven out of fourteen articles, according to which buccal mucosa was the predominant site, followed by gingiva, retromolar trigone, and others [20,21,24,30,33,34,35].

**Figure 2:**
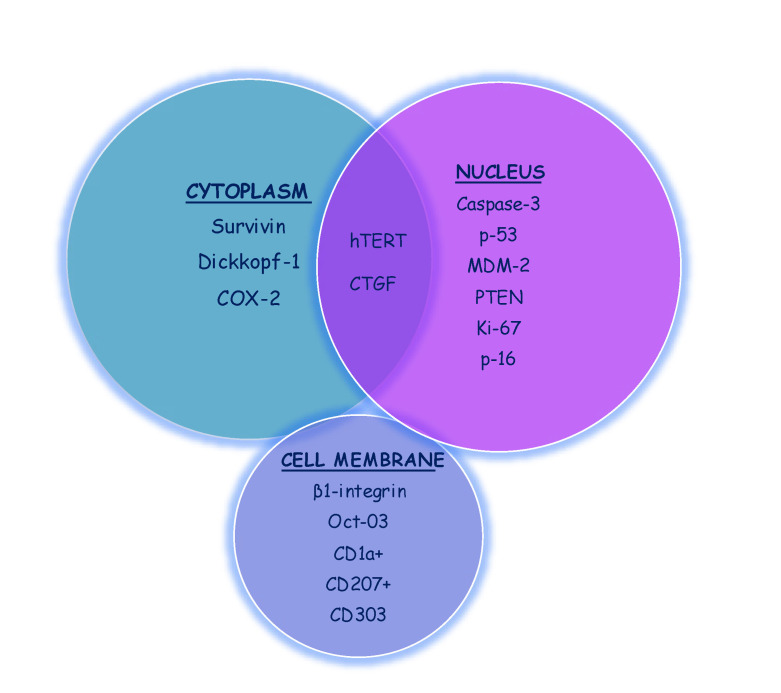
Cellular distribution of the epithelial markers.

We categorized the markers into epithelial markers and connective tissue markers as shown in Table 1 and Table 2. The epithelial markers were further sub-categorized as cytoplasmic, nuclear, and cell membrane markers (Figure 2).

#### 3.2.1. Epithelial Markers

Five studies evaluated five epithelial cytoplasmic markers [20,21,24,26,30], which included survivin [20], COX-2 [24], Dickkopf-1 [30], hTERT [21], and CTGF [26]. Tissue localization of survivin, COX-2, hTERT, and CTGF was restricted to basal and suprabasal layers of epithelium in the non-transformed group and invading islands in the transformed group. The staining intensity of all these markers was increased from the non-transformed to the transformed group. Similarly, a number of positive cases with these markers was also increased in the transformed group as compared to the non-transformed group. COX-2 was positive in 10 out of 20 (50%) patients in the non-transformed group and in 10 out of 10 (100%) patients in the transformed group [24]. Survivin was positive in 14 out of 50 (28%) patients in the non-transformed group and 50 out of 52 (96.15%) patients in the transformed group. Additionally, hTERT was positive in 14 out of 20 (70%) patients in the non-transformed group and 5 out of 5 (100%) patients in the transformed group. CTGF was positive in 35 out of 40 (87.5%) patients in the non-transformed group and in 10 out of 10 (100%) patients in the transformed group. Quantitative evaluation of immunoexpression demonstrated a higher value of survivin and Dickkopf-1, whereas a lower value of hTERT and CTGF was demonstrated in the non-transformed group as compared to the transformed group. The mean survivin score was 6.5 in the non-transformed group and 5.38 in the transformed group [20]. H-scores of Dickkopf-1 in the non-transformed group and the transformed group were 173.1 and 100.3, respectively [30]. The hTERT labeling score was 6.15 ± 1.981 in the non-transformed group and 7.2 ± 1.095 in the transformed group [21]. The quickscore of CTGF was 3.75 in the non-transformed group and 6.7 in the transformed group [26].

Eight studies [21,23,24,25,26,27,29,36] evaluated nine epithelial nuclear markers, such as caspase-3 [23], p53 [24,27], MDM2 [24], PTEN [25], CTGF [26], Ki-67 [29,36], p16 [36], hTERT [21], and CTGF [26]. Tissue distribution of only four markers, such as hTERT [21], CTGF [26], PTEN [25], and p53 [27], were compared between the two groups. All these four markers were restricted to basal and suprabasal layers of epithelium in the non-transformed group, whereas it was in the invading tumor cells in the transformed group. Similarly, comparative data on staining intensity were available for only four markers, among which, p53 [24], MDM2 [24], Ki67 [29,36], and hTERT [21] were more intensely expressed in the transformed group as compared to the non-transformed group, whereas PTEN [25] and Caspase-3 [23] expressions were less intense in the same group. The number of positive cases with five markers, such as hTERT, CTGF, Ki67, MDM2, and P53, was far more in transformed cases as compared to non-transformed OSMF. Additionally, hTERT was positive in 14 out of 20 (70%) patients in the non-transformed group and 5 out of 5 (100%) patients in the transformed group. In 9 out of 20 (45%) patients and in 4 out of 20 (20%) patients in the non-transformed group, positive immunoexpression of p53 and MDM2, respectively, was found, and both these markers had positive immunoexpression in 10 out of 10 (100%) patients in the transformed group [24]. No positive cases of PTEN expression were noted in either group [25]. In the study conducted by Trivedy et al. [27], 13 out of 21 (61.9%) patients in the non-transformed group and 2 out of 6 (33.33%) patients in the transformed group showed positive expression of p53. Yadahalli et al. [36] conducted a study, in which no immunoexpression of p16 was noticed in the non-transformed group as well as in the transformed group, and no immunoexpression of Ki-67 was seen in the non-transformed group, while positive immunoexpression of Ki-67 was seen in 10 out of 10 (100%) patients in the transformed group. Immunoexpression was quantified only in two markers, such as caspase 3 and Ki67. Mean score of the caspase-3-positive case was 8.93 ± 11.57 in the non-transformed group and 2.12 ± 3.575 in the transformed group [23]. The labeling indexes of Ki-67 were 28.23 ± 5.76 and 57.85 ± 8.51 in the non-transformed group and the transformed group, respectively, in study conducted by Gadbail et al. [29].

Three studies [22,33,35] were carried out on five membranous epithelial markers, such as β1 integrin [22], CD1a [35], CD207 [35], CD303 [35], and OCT-3 [33], out of which, three are dendritic cell markers [22]. Distribution of β1 integrin was mostly seen in the basal and suprabasal layer of epithelium in the non-transformed group and in peripheral and central cells of tumor islands in the transformed group [22]. The intensity of the immunoexpression of β1 integrin was found to be more intense in the transformed group as compared to the non-transformed group [22]. Positive β1 integrin was found in 55 out of 81 (67.9%) patients in the non-transformed group and 16 out of 16 (100%) patients in the transformed group [22]. The number of immunopositive cases with CD1a, CD207, or CD303, and stain intensity were not reported [35]. CD1a and CD207 were reportedly expressed in the basal cell layer in both groups [35]. The mean number of CD1a+ cells was higher in the non-transformed group in comparison to the transformed group (57 ± 42.97 and 40.11 ± 22.44OSMF-OSCC, respectively), but the difference was not statistically significant [35]. The mean number of CD207-positive cells was 35.67 ± 25.65 in the non-transformed group and 26.89 ± 26.15 in the transformed group; but again, the difference was not statistically significant [35]. Conversely, the mean of CD303-positive cells was lower in the non-transformed group (0.21 ± 0.58) in comparison to the transformed group (2.22 ± 2.49), although both were statistically insignificant [35]. Tissue distribution of OCT-3 and the number of immunopositive cases with OCT-3 were not reported [33]. OCT-3 integration optic density was significantly higher in the transformed group in comparison to the non-transformed group [33]. Intensity of OCT-3 immunoexpression increased from the non-transformed group to the transformed group [33].

#### 3.2.2. Connective Tissue Markers

Four studies [25,26,29,34] evaluated four connective tissue markers that included α-SMA [25,29], CTGF [26], CD105 [29], and lysyl oxidase [34]. In both groups, while cellular localization of α-SMA and CD105 is cytoplasmic, CTGF expression was observed in both the nucleus and cytoplasm. Lysyl oxidase was seen in the cytoplasm in the non-transformed group, and in the extracellular matrix in the transformed group. In the non-transformed group, α-SMA was seen around vessel walls in the myofibroblast, and throughout the stroma [25,29], CTGF distribution was seen around blood vessels and in skeletal muscles [26], and lysyl oxidase distribution was limited to the upper third of lamina propria [34]. In the transformed group, distribution of α-SMA was seen in myofibroblast in connective tissue stroma [25] and in neoplastic infiltrated islands [29], and lysyl oxidase distribution was noticed in stromal reaction of tumors directly adjacent to invading epithelium [34]. None of the connective tissue markers were differentially expressed in both groups, except CTGF and lysyl oxidase, while CTGF was not expressed in the non-transformed group, but strongly expressed in transformed group [26]. Expression of lysyl oxidase was more intense in the non-transformed group as compared to the transformed group [34]. There are no marked differences in the number of positive cases among the two groups, except lysyl oxidase, which was positive in 50% of non-transformed cases, whereas it was positive in 0% in transformed cases. Quickscore of CTGF was 4.03 and 6.7 in the non-transformed group and transformed group, respectively [26], as shown in Table 2.

**Figure 3:**
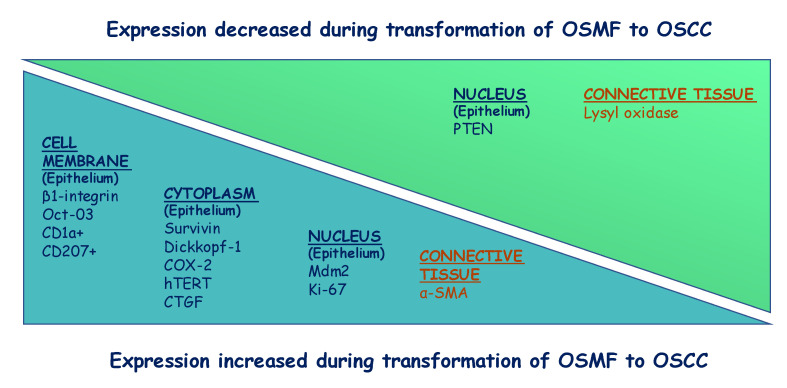
Summary of change in expression of markers during transformation of OSMF to OSCC.

To summarize, expression of β1 integrin [22], OCT-3 [33], CD1a [35], CD207 [35], survivin [20], Dickkopf-1 [30], COX-2 [24], hTERT [21], CTGF [26], MDM2 [24], Ki-67 [29], and α-SMA [25,29] were increased during transformation of OSMF to OSCC. Conversely, expression of PTEN [25] and lysyl oxidase [34] decreased during transformation of OSMF to OSCC, as shown in Figure 3.

### 3.3. Quality Assessment

Quality assessments of all the included studies were conducted as per Newcastle–Ottawa scale checklist for cross-sectional studies, based on three parameters: selection, comparability, and outcome. NOS score of each study was found to be more than six, as shown in Table 1 and Table 2.

### 3.4. Meta-Analysis

#### 3.4.1. Epithelial Markers

**Figure 4:**
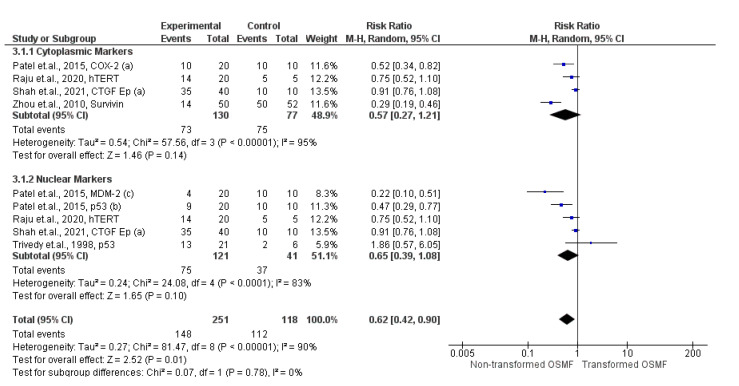
Forest plot showing comparison of epithelial markers between the non-transformed group and the transformed group [20,21,24,26,27].

Expression of epithelial markers was 38 times lower in the non-transformed group as compared to the transformed group (95% CI: 58% to 10%; *p* = 0.01; I^2^ = 90%). Further sub-group analyses of cytoplasmic and nuclear epithelial markers were conducted, which were inconclusive. Forest plots are shown in Figure 4.

#### 3.4.2. Cell Metabolism/Proliferation/Apoptosis Markers

**Figure 5:**
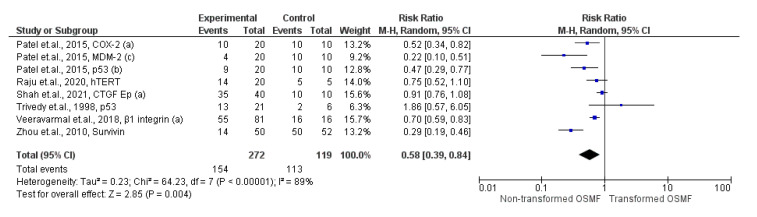
Forest plot showing comparison of cell metabolism/proliferation/apoptosis markers between the non-transformed group and the transformed group [20,21,22,24,26,27].

The expression of cell metabolism/proliferation/apoptosis markers was 42 times lower in non-transformed group as compared to transformed group (95% CI ranged from 61% to 16%; *p* = 0.004; I^2^ = 89%), as shown in Figure 5.

### 3.5. Publication Bias

We evaluated a small number of studies and observed a large difference in the sample size of individual studies. Therefore, Egger’s test was conducted to assess publication bias. Egger’s test was conducted on four studies that assessed epithelial cytoplasmic markers [20,21,24,26] and on five studies that assessed epithelial nuclear markers [21,24,26,27], as shown in Table 3.

### 3.6. Discussion

This systematic review and meta-analysis presents an extensive overview of qualitative and quantitative differences in the immunoexpression of tissue biomarkers in non-transformed and transformed OSMF. Qualitative features included the tissue distribution and intensity of immunoexpression, whereas quantitative features described the number of positive cases and immunohistochemical scores in the two groups. The list of biomarkers as found in this systematic review and meta-analysis would at least identify the potential IHC markers, which need to be studied in longitudinal studies.

Thirteen out of fourteen studies were conducted in southeast Asian countries [20,21,22,23,24,25,26,27,29,30,33,34,36], and only one was conducted in the South American population [35]. The high prevalence of OSMF in Southeast Asian countries [37] focuses OSMF related research in these countries. Male-to-female ratio in non-transformed OSMF was 4:1 [21,24,25,26,30,35], and in transformed OSMF was 6:1 [20,21,24,25,26,30,35]. Etiopathogenesis of OSMF is attributed to beetle nut chewing, which is more common among males [38]; thus, the male-to-female ratio in both study groups in the present systematic review is high. These fourteen studies compared the immunoexpression of nineteen biomarkers in two groups. Thirteen studies evaluated sixteen epithelial markers and four studies evaluated connective tissue markers. Epithelial markers were further subdivided into membranous, cytoplasmic, and nuclear markers based on cellular localization. Qualitative analysis of five cytoplasmic markers demonstrated that there is a progressive increase in β-integrin, Dickkopf-1, OCT-3, hTERT, and CTGF during transformation of OSMF. Meta-analysis also supported this by demonstrating a 41% less expression of survivin, COX-2, hTERT, and CTGF in the non-transformed group as compared to the transformed group (95% CI ranged from 63% to 79%; *p* = 0.31; I^2^ = 97%). Similarly, systematic review on ten nuclear markers demonstrated that there is a progressive increase in MDM2 and Ki-67 during transformation of OSMF. In addition to the qualitative analysis, meta-analysis also demonstrated 35 times lower expression of nuclear markers in the non-transformed group compared to transformed group cases (95% CI ranged from 61% to 8%; *p* = 0.10; and I^2^ = 83%). Owing to a similar increase in immunoexpression of p53 in oral lichen planus (OLP) associated OSCC compared to non-transformed OLP as reported by Valente et al. [39], we may hypothesize that p53 overexpression may be an indicator of the malignant transformation of OPMDs. A recent systematic review also supported the role of p53 in the malignant transformation of OPMDs [40]. In fact, p53 is also suggested as a prevalent biomarker in proliferative verrucous leukoplakia (PVL), having a high risk of malignancy [41]. As for MDM2, under normal conditions, SUMO-1 binds to it and prevents self-ubiquitination of MDM2, leading to controlling the p53 level too subsequently [42]. Supporting the present findings, Oliveira Alves et al. [43] demonstrated overexpression of MDM2 in OLP compared to normal mucosa. This may suggest a pro-transformation role of MDM2 in OPMDs. Further evaluation of SUMO-1 is required to elucidate the role of apoptotic markers in the malignant transformation of OPMDs. Ki-67 is known as a ubiquitous marker of cell proliferation. The evidence of the role of Ki-67 in the malignant transformation of leukoplakia [44,45], PVL [41], and OLP [46] is not sufficient as reported by few latest systematic reviews. However, the progressive overexpression in OPMDs and transformed cases of OSCC were observed in all these reports [41,44,45,46]. Therefore, in addition to the present findings, further exploration into the pro-transformation role of Ki-67 should be made. On the contrary, the immunoexpressions of PTEN and p16 were found to decrease with the progression of non-transformed group to transformed group; p16 is a tumor suppressor gene regulating the cell cycle. Loss of p16 expression is commonly observed in the process of carcinogenesis [47]. Although OSCC had no p16 immunoreactivity in comparison to 26.7% in OLP, the predictive role of p16 in the malignant transformation of OLP could not be assumed [48]. The meta-analysis of non-transformed and transformed cases expressing nuclear markers p53, hTERT, CTGF, and MDM2 demonstrated the 35 times lower expression in non-transformed OSMF compared to transformed OSMF. Although the result was found to be statistical insignificant (*p* = 0.10; I^2^ = 83%), there is supportive evidence of Ki-67, p53, and p16 being predictive markers of the malignant transformation in leukoplakia and other OPMDs [13,49,50,51].

The comparison between tissue distributions of seven cytoplasmic markers, such as survivin, hTERT, MT-1MMP, TIMP-1, TGF-β1, CTGF, and Dickkopf–1 is inconclusive because of inconsistent/inadequate reporting in two groups, except for CTGF and hTERT, which were localized to basal layers of epithelium in the non-transformed group, whereas they were distributed both in the basal layer and invading epithelial islands in the transformed group. The intensity of the immunostaining of survivin, COX-2, hTERT, and CTGF was seen to progressively increase from the non-transformed group to the transformed group. Therefore, it may be assumed that these molecules may play a role in the malignant transformation of OSMF. The comparison of quantitative findings revealed that immunoexpression of survivin, COX-2, hTERT, and TGF-β1 is 41% lower in non-transformed cases compared to transformed cases, though this result is inconclusive (*p* = 0.14, I^2^ = 95%).

The comparison between tissue distributions of 10 nuclear markers, such as p53, hTERT, caspase-3, TGF-β1, MDM2, PTEN, CTGF, Ki-67, p16, and BMI-1 was also inconclusive because of inadequate reporting. Tissue distribution of p53, hTERT, PTEN, MDM2, and CTGF was restricted to basal or/and suprabasal layers in the non-transformed group and epithelial islands in addition to basal layers in the transformed group. Intensity of immunoexpression of p53, MDM2, and Ki-67 was weak and strong in the non-transformed and transformed group, respectively, which may be interpreted as the involvement of these molecules in the malignant transformation of OSMF.

While evaluating five membranous markers, such as β-integrin, CD1a, CD207, CD303, and OCT-3, the tissue distribution of only β1 integrin was clearly reported as restricted to basal and suprabasal layers in the non-transformed group, while in the tumor islands in the transformed group. There was no difference in expression of CD1a and CD207 between two groups. Difference in intensity of immunoexpression was evident for only β1 integrin, which was more intense in the transformed group compared to the non-transformed group, which suggested a possible role of β1 integrin in the malignant transformation of OSMF. Contradicting lack of evidence on the role of β1 integrin was previously observed in the malignant transformation of oral dysplasia and leukoplakia [52,53].

Tissue distribution and intensity of four connective tissue markers, such as CD105, α-SMA, CTGF, and lysyl oxidase were compared between the two groups. Lysyl oxidase was found to be stronger in the non-transformed group compared to the transformed group. Immunoexpression of CTGF was found to be intense in the transformed group compared to the non-transformed group. There was no difference in immunoexpression of α-SMA between the two groups. Difference in tissue distribution of lysyl oxidase was clearly localized to the upper third of lamina propria in the non-transformed group and surrounding the invading epithelial cells in the transformed group. There may be several suggested mechanisms of lysyl oxidase mediated the malignant transformation of OSMF. First, excess extracellular matrix modification through cross-linking of collagen may stimulate invasion and metastasis [54]. Second, lysyl oxidase may regulate the cell signaling pathway by interacting and oxidizing other non-collagen proteins to modulate cancer progression [55,56,57]. The present finding on progressive increased immunoexpression of lysyl oxidase from non-transformed OSMF to transformed OSMF supported the role of lysyl oxidase in carcinogenesis and tumor progression in several organs, such as colorectal and esophageal cancer [58,59,60].

Among several mechanisms of the malignant transformation of OSMF alteration of oncogenes, tumor suppressor genes, DNA repair genes, and antiapoptotic genes and their products play the most significant role [61]. Although there are much primary research focused on identifying candidate genes predicting the malignant transformation of OSMF, this systematic review and meta-analysis, for the first time ever, compiled all possible IHC markers responsible for the malignant transformation of OSMF. The result would give a list of candidate markers, which can be prospectively evaluated for its malignant transformation potential. Presence of HLA-DR-positive cells and high CD4 to CD8 cells in OSMF were also shown to contribute towards the malignant transformation of OSMF [62]. However, this factor is beyond the scope of the present systematic review and meta-analysis. Therefore, further studies looking at all the possible mechanisms ensembled together would be appreciable. Although molecular events are happening in both epithelium and connective tissue, this systematic review and meta-analysis stressed the role of epithelial markers in the malignant transformation of OSMF.

As of now, the literature evidenced the biomarkers predicting the malignant transformation of oral leukoplakia, PVL, and OLP [40,41,44,45,46,52,53,63]. To the best of our knowledge, the present systematic review is the first of its kind in evaluating the potential of immunomarkers in predicting malignant transformation in OSMF. However, there are a few limitations of this systematic review and meta-analysis. First, because of a lack of prospective longitudinal studies, this research included only retrospective comparative cross-sectional studies. Second, the absence of sample calculations also influenced the result erroneously. The third limitation is attributed to the immunohistochemical technique, which produced only qualitative results in most of the articles, thereby making the analysis prone to subjective interpretations. Fourth, the present systematic review did not use strict statistical criteria for article inclusion.

There was a marked increase in the malignant transformation rate of OSMF to 5.5%, which necessitates the identification of IHC markers having the potential to predict the malignant transformation of OSMF [64]. The events listed in this systematic review and meta-analysis for their possible predictive role in the malignant transformation of OSMF are upregulation of COX2, hTERT, CTGF, survivin, MDM2, and p53. Furthermore, hTERT and survivin are apoptosis-inhibiting proteins. Cox-2 and MDM2 are the oncogenes and CTGF is a member of TGF- β family. Increased expression of p53 may be due to mutation in this tumor suppressor gene, resulting in accumulation of abnormal p53 protein. Due to the limited evidence available, immunohistochemical markers are rarely utilized in the clinical setting to predict the malignant transformation of OSMF. The markers identified in this study warrant further investigation to explore their clinical utility. Instead of delving into numerous markers without a strong basis for their use, it is advisable to further study the markers identified in this study to explore their clinical utility either individually or in combination.

## 4. Conclusions

This systematic review identified 19 immunohistochemical markers that were compared in the non-transformed and transformed OSMF. There may be a potential increase in the immunohistochemical expression of β1-integrin, OCT-3, CD1a, CD 207, survivin, Dickkopf-1, COX-2, hTERT, CTGF, MDM2, Ki-67, and α-SMA during the malignant transformation of OSMF. Conversely, downregulation of PTEN and lysyl oxidase may be responsible for the malignant transformation of OSMF. Upregulation of COX2, hTERT, CTGF, survivin, MDM2, and p53, and β1-integrin showed a promising role in the malignant transformation of OSMF. The high heterogeneity in this meta-analysis necessitates further exploration of these markers in well-designed prospective longitudinal studies. In fact, studying all these markers together to observe their combined effect may effectively predict the malignant transformation of OSMF. Moreover, these biomarkers should also undergo required validation procedures to determine their true predictive value in clinical settings.

## Figures and Tables

**Table 1:** Epithelial Markers.

Epithelial Markers	Cellular Distribution	Tissue Distribution		Intensity of Expression		No. of Positive Cases		IRS/Labeling Index/Quickscore/H-Score		NOS
		OSMF	OSMF + OSCC	OSMF	OSMF + OSCC	OSMF	OSMF + OSCC	OSMF	OSMF + OSCC	
Survivin [20]	Cytoplasm	Basal/parabasal and prickle cell layer	NA	Weak to moderate	Strong	14/50	50/52	6.5	5.38	8
Dickkopf-1 [30]	Cytoplasm	NA	NA	NA	Weak	NA	NA	6.7	100.3	6
COX-2 [24]	Cytoplasm	Basal and supra basal	Throughout epithelium and invading islands	Moderate to strong	Strong	10/20	10/10			
hTERT [21]	Cytoplasm and nucleus	Basal and suprabasal layers	Tumour islands	Moderate	Strong	14/20	5/5	6.5 ± 1.981	7.2 ± 1.095	6
CTGF [26]	Cytoplasm and nucleus	Basal layer of epithelium	epithelium as well as tumor islands	NA	Strong	35/40	10/10	3.75	6.7	7
Caspase-3 [23]	Nucleus	Basal	NA	Moderate	Weak	NA	NA	NA	NA	6
Ki-67 [36]	Nucleus	Basal cell layer	NA	Weak	Strong	0/10	10/10	NA	NA	6
Ki 67 [29]	Nucleus	NA	NA	Weak	NA	NA	NA	28.23 ± 5.76	57.85 ± 8.51	7
MDM2 [24]	Nucleus	NA	Epithelium and infiltrating islands	Weak	Strong	4/20	10/10	NA	NA	8
PTEN [25]	Nucleus	Basal and parabasal layer	Peripheral cells of tumour island	Weak	No	NA	NA	NA	NA	6
p16 [36]	Nucleus	All layers of epithelium	NA	NA	No	NA	0/10	NA	NA	6
p53 [24]	Nucleus	NA	Epithelium and infiltrating islands	Moderate	Strong	9/20	10/10	NA	NA	8
p53 [27]	Nucleus	Basal layer	Epihelial cells limited to few focal areas	NA	Strong	13/21	2/6	NA	NA	6
OCT-3 [33]	Cell Membrane	NA	NA	Weak	Strong	NA	NA	NA	NA	6
β1 integrin [22]	Cell membrane	Basal and suprabasal layers	Peripheral and central cells of tumour islands	Moderate and strong	Strong	55/81	16/16			6
CD1a [35]	Cell membrane	Basal cell layer	NA	NA	NA	NA	NA	NA	NA	7
CD207 [35]	Cell membrane	Basal cell layer	NA	NA	NA	NA	NA	NA	NA	7
CD303 [35]	Cell membrane	NA	NA	NA	NA	NA	NA	NA	NA	7

COX-2 = cyclooxygenase-2; CTGF = connective tissue growth factor; MDM2 = murine double minute 2; PTEN = phosphatase and tensin homolog; IRS = immunoreactivity score; NA = not available; and NOS = Newcastle–Ottawa scale.

**Table 2:** Connective tissue markers.

Connective Tissue Markers	Cellular Distribution		Tissue Distribution		Intensity of Expression		Number of Positive Cases		IRS/Quickscore		NOS
	Non-Transformed Group	Transformed Group	Non-Transformed Group	Transformed Group	Non-Transformed Group	Transformed Group	Non-Transformed Group	Transformed Group	Non-Transformed Group	Transformed Group	
α-SMA [25]	Cytoplasm	Cytoplasm	Around vessel walls in myofibroblast	Myofibroblast in connective tissue stroma	Weak	Weak	0/10	2/30	NA	NA	6
α-SMA [29]	Cytoplasm	Cytoplasm	Throughout the stroma	Neoplastic infiltrated islands	Weak	NA	NA	NA	NA	NA	7
CTGF [26]	Nucleus and cytoplasm	Nucleus and cytoplasm	around blood vessels and in skeletal muscles	NA	NA	High	40/40	10/10	4.03	6.7	7
CD105 [29]	Cytoplasm	Cytoplasm	NA	NA	NA	NA	NA	NA	NA	NA	7
LO [34]	Cytoplasmic process of fibroblast and extracellularly in upper third of lamina propria	Extracellular matrix (focaly in the stromal reaction of the tumour directly adjacent to invading epithelila islands	Upper third of Lamina Propria	Stromal reaction of tumour directly adjacent to invading epithelium	Moderate and strong	Weak	7/13	0/6	NA	NA	

α-SMA = α-smooth muscle actin; CTGF = Connective Tissue Growth Factor; LO = lysyl oxidase; NA = not available; IRS = immunoreactivity score; and NOS = Newcastle–Ottawa scale.

**Table 3:** Egger’s test of epithelial cytoplasmic markers and epithelial nuclear markers.

Egger’s Test (Epithelial Cytoplasmic Markers)			
	*p*-Value	CI (Lower Limit)	CI (Upper Limit)
**Epithelial Cytoplasmic Markers (*n* = 4)**	0.538	−5.878	6.760
**Epithelial Nuclear Markers (*n* = 5)**	0.940	−0.870	0.916

## Data Availability

Not applicable.
